# Influence of Peptide-Rich Nitrogen Sources on GAD System Activation and GABA Production in *Levilactobacillus brevis* CRL 2013

**DOI:** 10.3390/ijms27010082

**Published:** 2025-12-21

**Authors:** María Paulina Urquiza Martínez, Pablo G. Cataldo, Natalia Soledad Ríos Colombo, Pasquale Ferranti, Lucila Saavedra, Elvira M. Hebert

**Affiliations:** 1 Centro de Referencia para Lactobacilos (CERELA-CONICET), Chacabuco 145, San Miguel de Tucumán 4000, Argentina; purquiza@cerela.org.ar (M.P.U.M.); pcataldo@cerela.org.ar (P.G.C.); 2 APC Microbiome Ireland, University College Cork, T12 YT20 Cork, Ireland; nsoledadrioscolombo@ucc.ie; 3 Department of Agricultural Sciences, University of Naples Federico II, via Università 100, 80055 Portici, Italy; ferranti@unina.it

**Keywords:** *Levilactobacillus brevis*, GABA, lactic acid bacteria, glutamate decarboxylase, proteomics, nitrogen source, fermented foods, GABA synthesis

## Abstract

γ-Aminobutyric acid (GABA) is a bioactive metabolite valued in functional foods, but its microbial production is strongly influenced by nutrient availability. *Levilactobacillus brevis* CRL 2013 is an efficient GABA producer; however, its biosynthesis depends on culture medium composition. In this study, integrated physiological, proteomic, and transcriptional analyses were applied to assess the influence of nitrogen source composition and concentration on GABA production. No extracellular GABA was detected in a chemically defined medium containing all amino acids and glutamate (CDMg), whereas supplementation with yeast extract or Casitone restored high-level production. The highest GABA accumulation (~250 mM) was obtained in CDMg supplemented with 1% yeast extract or 2% Casitone, and a clear peptide dose-dependent effect was observed. In contrast, other protein hydrolysates or free amino acids alone did not stimulate GABA synthesis. Proteomic analysis revealed overexpression of the key enzyme GadB and changes in nucleotide and fatty acid pathways. Transcriptional analysis confirmed that peptide supplementation was accompanied by increased transcription of the *gadRCB–gltX* operon, in agreement with GABA accumulation. Overall, these results demonstrated that peptide composition and availability are critical determinants of GABA biosynthesis in *Lv. brevis* CRL 2013and provide a basis for optimizing peptide-based media to enhance GABA formation in food fermentations.

## 1. Introduction

Lactic acid bacteria (LAB) are microorganisms widely used in food fermentation due to their generally recognized as safe (GRAS) status, metabolic versatility, and contribution to the organoleptic and technological properties of fermented products [1]. Beyond their traditional role as starters or adjunct cultures, several LAB strains are recognized for producing metabolites with potential health benefits, thereby linking food fermentation with nutrition and well-being [2,3]. Among the metabolites produced by LAB during food fermentation, γ-aminobutyric acid (GABA) has received increasing attention due to its dual technological and functional relevance [4,5,6]. GABA is a non-proteinogenic amino acid synthesized by certain LAB through the glutamate decarboxylase (GAD) system, which converts glutamate into GABA while consuming intracellular protons [4]. This reaction contributes to cytoplasmic pH homeostasis under acidic conditions and enhances bacterial persistence in food environments [7,8]. Beyond its microbial function, dietary GABA has been associated with several physiological effects in humans, including anti-inflammatory, hypotensive, anxiolytic, antioxidant, and antidiabetic activities [5,9,10,11,12]. For these reasons, GABA-producing LAB have gained increasing interest in the context of functional foods and microbial physiology research [4,13,14].

Fortification of foods and beverages with GABA is increasingly explored due to its reported health benefits [15,16,17]. However, in several regions, including Argentina and the European Union, GABA is not currently authorized as a direct food additive [18]. Consequently, in situ GABA formation by food-grade microorganisms during fermentation is the preferred technological route for generating GABA-enriched products. This highlights the importance of optimizing production in strains with high biotechnological potential, such as *Levilactobacillus brevis* CRL 2013.

In food fermentation ecosystems, the composition and quality of nitrogen sources derived from raw materials such as milk, cereals, or vegetables play a decisive role in shaping LAB physiology and metabolite production [19]. Natural food matrices provide complex mixtures of peptides and amino acids originating from proteins such as casein, gluten, or plant proteins, which can differentially modulate microbial stress responses and metabolite biosynthesis, including GABA production. GABA biosynthesis by LAB is mediated by the GAD system, composed of the decarboxylases (GadA and/or GadB), the glutamate/GABA antiporter GadC, and the transcriptional regulator GadR [4,20]. This system catalyzes GABA formation while contributing to acid resistance by maintaining cytoplasmic pH and promoting ATP generation under acidic stress [21,22].

Among LAB, the food-associated strain *Levilactobacillus brevis* CRL 2013 is one of the most efficient microbial GABA producers identified to date [20]. However, its productivity is highly dependent on the composition of the growth medium, particularly the nitrogen source [20]. Previous studies showed that *Lv. brevis* CRL 2013 was unable to synthesize GABA in a chemically defined medium (CDM) containing all amino acids and monosodium glutamate (CDMg), whereas supplementation with yeast extract (YE), a complex nitrogen-rich source containing peptides as well as vitamins, minerals, nucleotides, and growth factors, restored high-level GABA production (~260 mM) and induced overexpression of *gadB* and *gadR* genes [20]. The influence of nitrogenous components on the GAD system is not exclusive to *Lv. brevis*. For instance, Paudyal et al. [23] reported that supplementing a CDM with nitrogen sources such as peptone or tryptone significantly activated GABA synthesis in *Listeria monocytogenes*. More recently, a large-scale screening of 132 *Lactococcus* strains confirmed that GABA production is a highly strain-dependent trait [24]. In this study, the *Lactococcus lactis* NCDO 2118 strain reached a conversion rate of only ~11% in a medium containing YE, a value notably lower than the high-efficiency conversion previously observed for *Lv. brevis* CRL 2013 [20]. These observations underscore the importance of understanding how specific nitrogenous fractions, particularly peptides, modulate the GAD system to maximize the biotechnological potential of high-producing strains. These observations underscore a critical discrepancy: GABA biosynthesis in CRL 2013 is not limited by general nitrogen sufficiency, but rather by specific components present in complex nitrogen sources. While peptides are considered the most likely contributors, their precise identity, or that of other potential cofactors, remains unresolved. To date, most studies have focused on glutamate concentration and pH as primary determinants of GABA production, leaving the specific role of peptide composition and size largely unexplored [4,24,25].

In this context, the present study examines the influence of different nitrogen sources, varying in peptide abundance and complexity, on the physiological, proteomic, and transcriptional responses associated with GABA production in *Lv. brevis* CRL 2013. Specifically, this work aims to (i) compare the impact of peptide-rich hydrolysates versus amino-acid–based nitrogen sources on GABA production; (ii) evaluate whether peptide concentration or peptide-size fractions affect the magnitude of GABA synthesis; and (iii) characterize the molecular signatures associated with the activation of the *gadRCB–gltX* transcriptional unit under different nitrogen conditions. By integrating these datasets, the study identifies the nitrogen-source conditions under which peptide supplementation is associated with increased GAD system activity, providing a basis for future physiological investigations.

## 2. Results

### 2.1. Effect of Nitrogen Source Supplementation on GABA Production and pH

Recent studies demonstrated that *Lv. brevis* CRL 2013 was unable to synthesize GABA in a chemically defined medium containing all amino acids and monosodium glutamate (CDMg) as the precursor [20]. In contrast, supplementation with yeast extract (YE) restored GABA production to levels comparable to those obtained in MRS medium. To rule out simple nutritional deficiencies, CDMg was supplemented with a threefold excess of amino acids and vitamins. No GABA production was detected under these conditions and growth and pH values remained similar to those observed in CDMg.

The effect of various complex nitrogen sources of both animal and plant origin, including vegetable peptone (VP) and different casein digests, on the growth, pH, and GABA production by *Lv. brevis* CRL 2013 was assessed (Table 1). Nitrogen sources with varying degrees of hydrolysis were tested: Tryptone (trypsin-hydrolyzed casein), Casitone (BD Bacto™, Difco pancreatic-hydrolyzed casein, containing 82% peptides and 18% free amino acids, [26]), and Casamino acids (acid hydrolysate of casein composed mainly of free amino acids).

**Figure 1:**
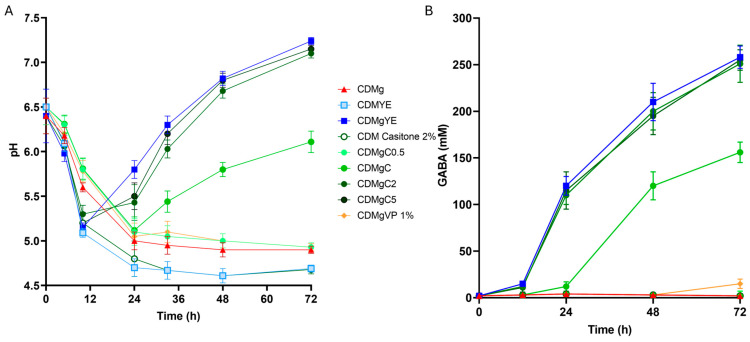
Growth medium acidification (**A**) and GABA production (**B**) by *Lv. brevis* CRL 2013 in CDM or CDMg supplemented with different nitrogen sources. CDMg, chemically defined medium containing monosodium glutamate; CDMYE, CDM without glutamate supplemented with 1% yeast extract; CDMgYE, CDMg supplemented with 1% yeast extract; CDM Casitone 2%, CDM without glutamate supplemented with 2% Casitone; CDMgC0.5, CDMg supplemented with 0.5% Casitone; CDMgC, CDMg supplemented with 1%; CDMgC2, CDMg supplemented with 2% Casitone; CDMgC5, CDMg supplemented with 5% Casitone; CDMgVP, CDMg supplemented with 1% vegetable peptone. The pH profile observed in CDMgC0.5 was similar to those obtained in CDMg supplemented with 1% tryptone and 1% casamino acids, none of which supported detectable GABA production. Data are expressed as means ± SD from at least three independent biological replicates.

Supplementation of CDMg with 1% (*w*/*v*) nitrogen sources enhanced bacterial growth, increasing both final biomass and specific growth rate (Table 1). However, only YE and Casitone induced a significant increase in medium pH, detectable from 12 h and 24 h, respectively (Figure 1). This pH increase was concomitant with extracellular GABA accumulation (Figure 1).

The conversion of glutamate to GABA by glutamate decarboxylase consumes protons and releases CO_2_; this proton consumption, together with the glutamate/GABA antiport system, contributes to an increase in extracellular pH [27]. When Casitone or YE were added without the glutamate precursor, neither GABA production nor pH increase was observed, confirming the strict requirement of glutamate for GABA accumulation (Figure 1). VP supplementation resulted in only a slight increase in GABA levels (~15 mM) without pH change, while Tryptone and Casamino Acids supported growth but did not induce GABA synthesis (Table 1). For CDMgYE, CDMg containing 2% Casitone (CDMgC2) and 5% Casitone (CDMgC5), a measurable GABA accumulation was already detected from 12 h of incubation, coinciding with pH increase. In CDMgC, both GABA production and pH shift appeared later, becoming evident at 24 h. In CDMg, no GABA was detected at any time point and pH remained acidic. These time-resolved data confirm that medium alkalinization is tightly coupled with GABA production kinetics.

Since sustained GABA production was selectively observed with Casitone (Table 1), the effect of its concentration (0, 0.5, 1.0, 2.0, and 5.0%) in CDMg was evaluated. No GABA production or pH increase was detected at 0.5% Casitone. GABA synthesis became detectable at 1%, reached its maximum at 2%, and showed no further increase at 5%. Increasing Casitone levels from 1% to 2% resulted in proportional increases in both GABA production and medium pH, reaching values comparable to those observed in MRS or CDMg supplemented with 1% YE (Figure 1). This dose-dependent response indicates that peptide-rich nitrogen concentration is the rate-limiting factor for GAD system induction and final GABA yield.

### 2.2. Effect of Peptide Size and Defined Peptides on GABA Biosynthesis

Casitone, a pancreatic digest of casein, contains a broad range of peptide fragments that are not fully hydrolyzed into free amino acids, unlike acid hydrolysates. The molecular weight distribution of Casitone shows that approximately 50% of the components are <500 Da, while only ~10% are between 2 and 5 kDa and negligible amounts exceed 5 kDa (according to manufacturer data sheet). To support the expected peptide-size distribution present in Casitone, an in silico digestion of bovine caseins (αS1-, αS2-, β- and κ-caseins) was performed using proteases with cleavage specificities equivalent to those used for industrial pancreatic digests (trypsin-, chymotrypsin- and carboxypeptidase-like rules). This computational analysis confirmed that casein hydrolysis generates a broad mixture of short peptides predominantly below 2000 Da, in agreement with the manufacturer’s molecular-weight profile of BD Bacto™ Casitone (with >70% of peptides <2000 Da).

To investigate whether peptide size contributes to GAD system activation, Casitone was fractionated into low–molecular mass (<3 kDa, LMMP) and high–molecular mass (>3 kDa, HMMP) fractions. The addition of LMMP fraction to CDMg at levels equivalent to Casitone 2% (*w*/*v*) partially restored GABA production (Table 1). In contrast, the HMMP fraction generated only trace amounts of GABA, approximately ten-fold lower than the LMMP fraction, indicating that short peptides are markedly more effective than longer peptides in supporting GAD activation (Table 1).

### 2.3. Proteomic Profiling of Lv. brevis CRL 2013 Under Different Nitrogen Supplementation Conditions

To investigate the molecular basis underlying the differences in GABA production, comparative proteomic analyses were performed under three conditions: CDMg (no GABA production), CDMgC (1% Casitone, intermediate GABA production), and CDMgYE (1% YE, maximal GABA production).

#### 2.3.1. Principal Component Analysis (PCA)

**Figure 2:**
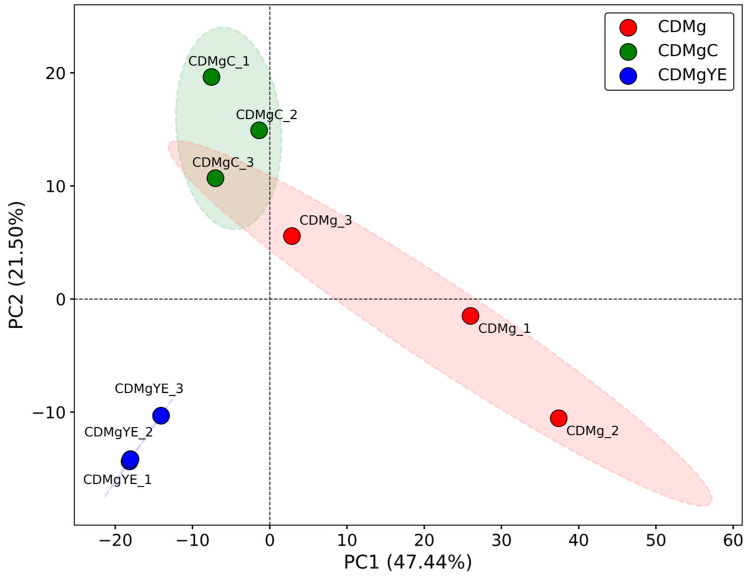
Principal component analysis (PCA) of proteomic profiles of *Lv. brevis* CRL 2013 under different nitrogen supplementation conditions. The first two principal components explained ~69% of the total variance (PC1: 47.4%, PC2: 21.5%). Each point represents a biological replicate: CDMg (red), CDMgC (green), and CDMgYE (blue). Shaded ellipses represent 95% confidence intervals.

**Figure 3:**
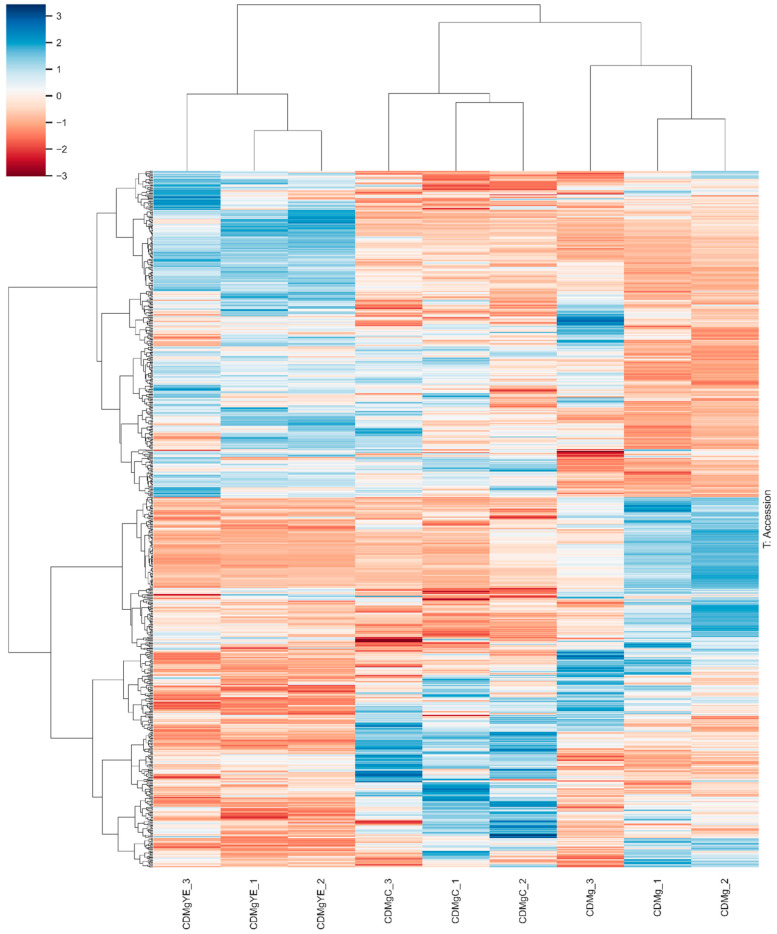
Hierarchical clustering heat map of quantified proteins in *Lv. brevis* CRL 2013 grown in CDMg, CDMgC and CDMgYE. Each column represents an independent biological replicate. Protein abundance values are shown as Z-scores, with red indicating lower and blue indicating higher relative abundance compared to the mean. Replicates group according to nitrogen supplementation, with CDMgYE forming a distinct cluster, CDMgC occupying an intermediate position, and CDMg samples clustering independently. The analysis confirms that each nitrogen condition generates a characteristic global proteomic profile.

PCA reduced the dimensionality of the dataset and captured the global variance structure across experimental conditions (Figure 2). The first two principal components explained ~69% of the total variance (PC1: 47.4%, PC2: 21.5%). Samples clustered into three well-defined groups: CDMgYE at the negative end of PC1, CDMg at the positive end, and CDMgC occupying an intermediate position, consistent with the gradient observed in physiological responses and GABA production. CDMg replicates displayed greater dispersion than CDMgC and CDMgYE, with one replicate (CDMg_3) positioned further from the group centroid; however, hierarchical clustering (Figure 3) confirmed that it remained within the CDMg cluster.

These multivariate analyses demonstrate robust separation between conditions, with CDMgYE and CDMg forming opposite extremes and CDMgC representing a transitional state between them. Statistical analyses supported these observations: PERMANOVA showed a significant global effect (pseudo-*F* = 4.39, *p* = 0.005), ANOSIM confirmed strong separation (*R* = 0.786, *p* = 0.006), and PERMDISP indicated no differences in dispersion (*F* = 0.233, *p* = 0.767).

#### 2.3.2. Hierarchical Clustering Analysis

Hierarchical clustering analysis of all quantified proteins revealed a clear and reproducible separation among the three nitrogen conditions (Figure 3). The heatmap, based on Z-scored protein abundances across all quantified proteins, showed that biological replicates clustered tightly, confirming high internal consistency of the proteomic dataset.

CDMgYE samples grouped together in a well-defined cluster, reflecting a characteristic global proteomic state under yeast extract supplementation. CDMgC replicates formed a distinct cluster positioned between CDMgYE and CDMg, consistent with their intermediate physiological behavior. In contrast, CDMg samples clustered separately, showing a proteomic profile markedly different from the peptide-supplemented conditions.

This hierarchical structure mirrors the experimental gradient previously observed (CDMg, CDMgC and CDMgYE), indicating that nitrogen source composition drives broad proteomic divergence across conditions.

#### 2.3.3. Distribution and Comparative Expression of DEPs

**Figure 4:**
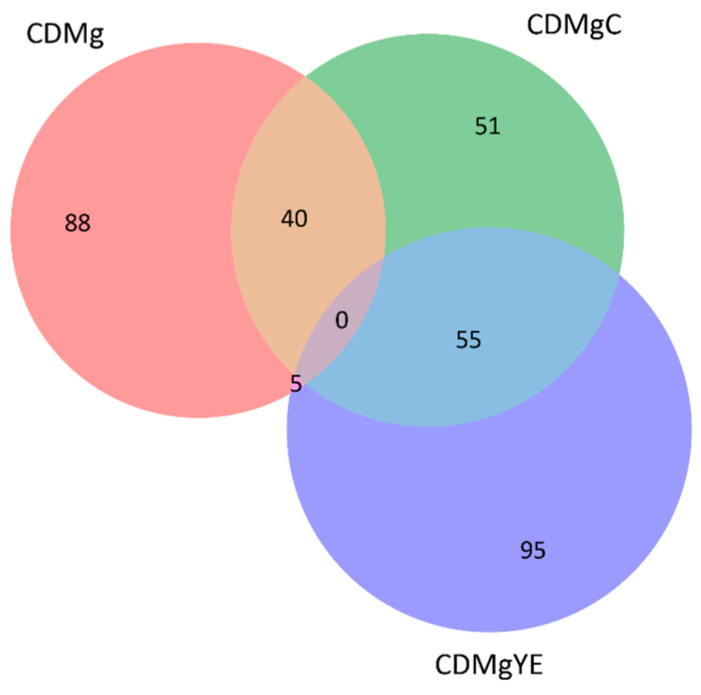
Venn diagram of differentially expressed proteins (DEPs) overexpressed in *Lv. brevis* CRL 2013 under different nitrogen supplementation conditions: CDMg, CDMgC and CDMgYE. Numbers indicate proteins showing significantly higher expression in one condition relative to the other two but not necessarily absent from them. DEPs were defined using Student’s *t*-test (*p* < 0.05) and a minimum relative expression change of 2-fold. The diagram was generated in Python (version 3.9; https://www.python.org).

A Venn diagram visualized differentially expressed proteins (DEPs) identified by Benjamini–Hochberg FDR–corrected Student’s *t*-tests (FDR < 0.05) combined with a ≥2-fold change threshold (Figure 4). A total of 88, 51 and 95 proteins showed higher abundance in CDMg, CDMgC and CDMgYE, respectively, relative to the other two conditions. Additional overlaps included 40 proteins shared between CDMg and CDMgC, 5 between CDMg and CDMgYE, and 55 between CDMgC and CDMgYE. In concordance with their distinct physiological profiles, no proteins were simultaneously overexpressed across all three conditions.

Relative to CDMg, CDMgC showed 106 proteins at higher abundance; 51 of these were exclusive to CDMgC, while 55 were shared with CDMgYE. These distributions indicate that CDMgC is proteomically closer to CDMgYE than to CDMg, consistent with physiological GABA-production patterns. In addition to DEPs, we also identified proteins that were exclusively detected in one or two conditions but were completely absent in the others.

Five proteins (Q03RV4, Q03Q29, Q03R97, Q03RW3, Q03P45) were detected exclusively under peptide-supplemented conditions (CDMgC and CDMgYE) and were not quantified in any replicate of CDMg (Table S1). In the LFQ dataset, these proteins appeared as Not a Number (NaN) across all CDMg samples, indicating that their abundance was below the detection threshold rather than completely absent under this condition. These included FabD (malonyl CoA-acyl carrier protein transacylase), BirA (biotin carboxyl-carrier protein ligase), PduB (propanediol utilization protein), MecA (adapter protein), and endoglucanase (LBR_02445). The detection of some of these proteins only under peptide-supplemented conditions aligns with the enhanced metabolic activity observed in CDMgC and CDMgYE, as these functions are commonly associated with accelerated growth and increased biosynthetic demand in LAB.

Conversely, a single protein, an RNA-binding protein with an S1 domain (Q03R92), was detected only in CDMg. This protein displayed quantifiable LFQ values in all CDMg replicates but fell below detection limits in peptide-supplemented cultures. Increased abundance of S1-domain proteins has been associated with reduced translational efficiency in other bacteria [28], which may be consistent with the lower growth rate observed in CDMg compared with peptide-rich media

**Figure 5:**
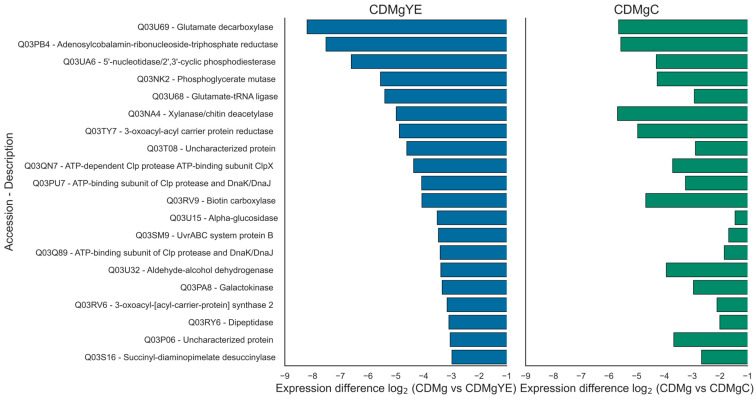
Differential expression of shared proteins between CDMgYE and CDMgC relative to CDMg. From the 55 proteins overexpressed in both CDMgYE and CDMgC relative to CDMg, the 20 most strongly upregulated in CDMgYE were selected (left blue panel). Their corresponding expression levels in Casitone (CDMgC) are shown for comparison (right green panel). Proteins are ranked according to their mean expression differences (log2) in CDMgYE. The most overexpressed was GadB (Q03U69, glutamate decarboxylase), directly involved in GABA synthesis. Bars represent mean log2 expression differences from three independent biological replicates.

Focusing on the core GABA biosynthetic machinery, GadB (Q03U69) was the most overexpressed protein across all peptide-supplemented conditions (CDMgC and CDMgYE). GltX (Q03U68), the glutamyl–tRNA ligase encoded within the same transcriptional unit, was also strongly upregulated (Figure 5). Both GadB and GltX proteins displayed expression trends proportional to GABA accumulation. In contrast, GadA (Q03PG2), another glutamate decarboxylase enzyme located outside the *gadRCB* locus, was detected under all conditions but showed no significant changes in abundance.

Beyond the GAD system, peptide supplementation induced a coordinated metabolic adaptation (Figure S1 and Table S1). Upregulated pathways included nucleotide metabolism (ribonucleoside-triphosphate reductase, 5′-nucleotidase), fatty acid synthesis (acetyl-CoA carboxylase, FabF), and protein quality control (ClpP, ClpX, ClpE), in concordance with accelerated growth under nitrogen-rich conditions.

In addition, Casitone supplementation led to an induction of the Opp oligopeptide transport and peptide-hydrolyzing systems (Table S2). Several components of the oligopeptide transporter system Opp showed upregulation, including OppA (3.83-fold), OppD (3.51-fold), OppF (2.89-fold), and OppB (1.94-fold). In parallel, intracellular peptidases displayed substantial increases in abundance, such as aminopeptidase Q03N78 (5.23-fold), dipeptidase Q03RY6 (4.01-fold), the neutral endopeptidase Q03PC0 (4.00-fold), and two additional dipeptidases (2.42–2.66-fold). These findings suggest that CDMgC promotes both enhanced oligopeptide uptake and intensified intracellular peptide turnover, a pattern not observed under CDMg. Although the genome encodes a predicted proton-motive-force di-/tripeptide transporter (DtpT, Q03SY7), this protein was not detected, likely due to its membrane localization and the intracellular extraction methods used.

Together, these results demonstrate that peptide supplementation induces a coordinated metabolic adaptation in *Lv. brevis* CRL 2013, encompassing enhanced peptide uptake and processing, nucleotide metabolism, fatty acid synthesis, protein quality control, and activation of the GAD pathway.

### 2.4. Transcriptional Analysis of GABA-Related Genes by RT-qPCR

**Figure 6:**
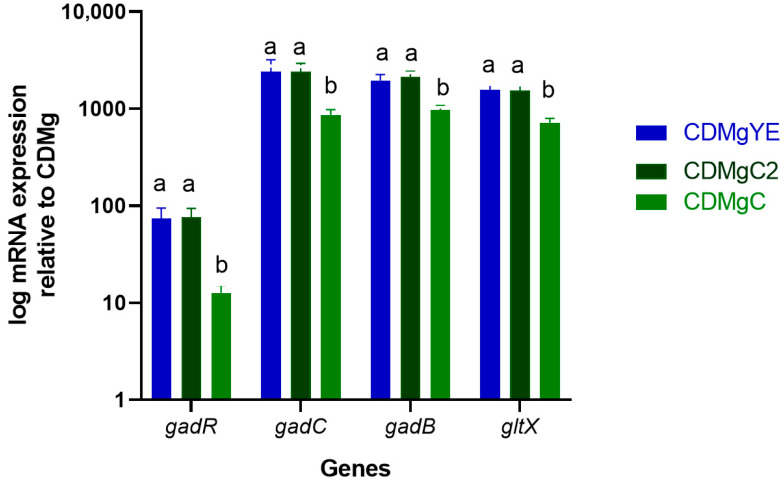
Relative expression of *gadR*, *gadC*, *gadB*, and *gltX* genes in *Lv. brevis* CRL 2013 grown in CDMgC (1%), CDMgC2 (2%), and CDMgYE, using CDMg as the reference condition. Gene expression was quantified by RT-qPCR and normalized to the reference genes *recA* and *rpoD*. Data are shown as log-transformed mRNA expression levels relative to CDMg (set to 1). Bars represent mean ± SD from three independent biological replicates. For each gene, bars that do not share a letter differ significantly (*p* < 0.05; one-way ANOVA followed by Tukey’s test).

To validate proteomic results and establish the correlation between gene expression and GABA production, the expression of key GAD operon genes (*gadR*, *gadC*, *gadB*, and *gltX*) was quantified by RT-qPCR in cells grown in CDMg, CDMgC (1%), CDMgC2 (2%), and CDMgYE (Figure 6). Using CDMg as the reference condition, all four genes were significantly upregulated in peptide-supplemented media. Transcript levels in CDMgC (1%) were intermediate between CDMg and CDMgYE, according to the GABA production observed. Importantly, when Casitone was increased to 2% (CDMgC2), gene expression levels reached those of YE supplementation, consistent with the comparable GABA concentrations measured physiologically in those two conditions. These transcriptional results confirm that the expression of the GAD operon genes is directly influenced by the composition and peptide content of the culture medium. In contrast, *gadA* showed no significant changes in expression, consistent with proteomic data indicating stable protein abundance across conditions. The strong concordance between gene expression, GadB protein levels, and GABA accumulation provides conclusive evidence that GAD regulation is driven by peptide composition and concentration in the medium.

## 3. Discussion

Understanding how nitrogen availability shapes GABA biosynthesis in food-associated LAB is essential for designing functional fermentations enriched in bioactive metabolites. This study provides new regulatory insights into the nitrogen source-dependent GABA biosynthesis in *Lv. brevis* CRL 2013. Our integrated physiological, proteomic, and transcriptional analyses reveal that nitrogen source composition, rather than overall nitrogen availability, acts as a key determinant factor governing the GAD system and triggering a broader, coordinated metabolic reprogramming. The multivariate PCA reinforced these results, demonstrating a clear physiological and proteomic gradient in the bacterial response, with the non-GABA producer CDMg and the maximal GABA producer CDMgYE occupying opposite extremes, while CDMgC consistently represented an intermediate transitional state. This gradient links nitrogen source quality to the magnitude of GABA biosynthesis. The inability of *Lv. brevis* CRL 2013 to produce GABA in CDMg, even after a threefold increase in free amino acids and vitamins, indicates that the absence of specific nitrogen-derived regulatory compounds, rather than a quantitative amino acid limitation, represents the main factor limiting GABA production. Restoration of GABA synthesis by YE and Casitone, but not by Tryptone, Casamino acids, or vegetable peptone, reinforces the hypothesis that peptide complexity, rather than total nitrogen availability, acts as a regulatory cue for GAD activation.

Casitone, a pancreatic digest of casein containing ~82% peptides and ~18% free amino acids [26], was the only nitrogen source tested besides YE that supported sustained GABA production. However, when Casitone was provided at 1% in CDM without glutamate supplementation, neither extracellular GABA accumulation nor an increase in medium pH was observed, confirming that glutamate is indispensable both as a substrate for the GAD pathway and as a contributor to intracellular pH homeostasis. This observation is consistent with food fermentation ecosystems, where the coexistence of peptides and glutamate-rich matrices determines whether GABA accumulation occurs [4,5,22]. This dependency highlights the interplay between nutrient composition and microbial physiology that governs functional metabolite synthesis in complex food matrices.

To further examine whether peptide size contributes to GABA induction, Casitone was fractionated into low–molecular mass peptides (<3 kDa, LMMP) and high–molecular mass peptides (>3 kDa, HMMP). The LMMP fraction partially restored GABA production, whereas HMMP did not, indicating that short peptides are more effective than longer ones in supporting GAD activation. However, neither fraction alone reproduced the full response observed with unfractionated Casitone, suggesting that peptide complexity or the combined action of multiple peptide species may be required to generate the intracellular conditions associated with GAD induction.

At the molecular level, proteomic and transcriptional analyses showed a strong, selective induction of the *gadRCB-gltX* operon under peptide supplementation, which correlated with GABA accumulation. GadB was the most strongly overexpressed protein, reaching 294-fold in CDMgYE and ~50-fold in CDMgC. Although GltX does not directly participate in GABA synthesis, its co-localization in the same operon as *gadB* explains its parallel overexpression [20,22]. In contrast, GadA abundance remained constant, consistent with its non-regulatory role.

This selective induction pattern reflects species-specific regulatory architecture. For instance, *Listeria monocytogenes* encodes several GAD isoenzymes (GadD1, GadD2, GadD3), only GadD2 responds to peptides, whereas in *Lv. brevis* CRL 2013, GadB (but not GadA) is peptide-responsive [7]. Despite these differences, the common principle across these Gram-positive bacteria is that nitrogen source quality, rather than abundance, modulates GABA production and acid stress adaptation pathways [7]. The correlation between extracellular GABA accumulation and medium alkalinization also supports the established role of the GAD system as an acid resistance mechanism [8,25,29].

The role of peptide transport systems provides further context for peptide-dependent GAD activation. Genome analysis of *Lv. brevis* CRL 2013 reveals two oligopeptide ABC transporter operons (*oppDFBCA* and *optABCDF*) and the PMF-dependent di-/tripeptide transporter *dtpT* [20]. Proteomic data showed induction of the Opp system in Casitone-supplemented cultures (OppA, OppD, OppF, OppB), together with increases in aminopeptidases and dipeptidases (Table S2). This combined upregulation indicates enhanced oligopeptide uptake and intracellular turnover in CDMgC, establishing conditions for GAD activation. DtpT was not detected, likely due to its membrane localization and the intracellular extraction method used.

In addition to the transport-associated responses, five proteins detected exclusively under peptide-supplemented conditions were consistently linked to the distinct physiological profile observed. FabD (ACP S-malonyltransferase) and BirA (biotin-protein ligase), both involved in fatty acid biosynthesis, were detected only in CDMgC and CDMgYE, suggesting increased membrane biogenesis that supports rapid growth and helps stabilize intracellular proton homeostasis during GAD-mediated alkalinization. This observation is structurally supported by the functional pathway in which *fabD*, *fabH*, and *fabG* genes encode malonyltransferase, 3-oxoacyl-ACP synthase, and reductase, respectively, contributing to lipid metabolism [30]. Enhanced lipid turnover would help maintain proton gradients by supporting membrane integrity and energy-dependent H^+^ extrusion [30]. BirA activates biotin-dependent carboxylases required for fatty acid and branched-chain amino acid synthesis. Its selective detection suggests reinforcement of membrane lipid metabolism and nucleotide biosynthesis to support increased precursor supply during peptide-stimulated growth.

PduB, annotated as a bacterial microcompartment shell protein, was also detected exclusively under peptide-supplemented conditions. Although PduB participates in microcompartment assembly in organisms such as *Salmonella enterica* [31], its physiological role in lactobacilli has not been characterized and no functional connection to nitrogen metabolism or GABA synthesis has been demonstrated. Therefore, in the context of this study, its presence is interpreted conservatively as part of the broader proteomic remodeling induced by peptide-rich media, rather than as evidence of microcompartment activation or functional involvement in the observed phenotype.

MecA, an adaptor of the ClpC/P protease system involved in proteostasis and general stress responses [32], was also selectively detected, likely indicating increased protein-quality-control demands during rapid growth and high GAD activity. Finally, LBR_02445, annotated as an endoglucanase based on sequence homology, represents a potential cell-wall-associated hydrolase. In Gram-positive bacteria, such enzymes often contribute to cell-envelope remodeling, modulating wall porosity and mechanical flexibility [33]. Such remodeling may facilitate nutrient accessibility and uptake functions compatible with the increased metabolic demands of active GABA production. However, the specific biochemical activity and physiological role of LBR_02445 have not yet been experimentally validated.

Together, these findings allow us to outline a plausible mechanistic framework for peptide-dependent GAD activation in *Lv. brevis* CRL 2013. Although the present study was not designed to experimentally dissect signaling pathways, the convergence of physiological, proteomic, and transcriptional results supports several non-exclusive mechanisms that may collectively explain the stimulation of GABA synthesis observed under peptide supplementation. Oligopeptides are imported with greater efficiency than free amino acids in LAB, and their intracellular hydrolysis by peptidases markedly increases the pools of amino acids [34,35]. Then, peptide catabolism accelerates growth and lactic acid formation, generating micro-acidic conditions which activate *gadR*-*CB* operon transcription as part of the acid tolerance response in Gram-positive bacteria [36]. Finally, peptides may also exert regulatory functions beyond their nutritional role [37]. In several Gram-positive organisms, Opp-mediated peptide uptake is interconnected with global nitrogen and stress-response pathways [38]. Although *Lv. brevis* lacks a well-characterized peptide-sensing regulator analogous to CodY or other nitrogen-responsive systems [19,35], the coordinated induction of *opp* transporters, peptidases, and *gadR* in our dataset suggests that intracellular peptide availability may indirectly modulate transcription of the *gadRCB-gltX* operon through broader stress or nitrogen regulatory networks. Thus, these results support a model in which peptides function not only as nitrogen sources but also as metabolic, regulatory, and physicochemical cues that collectively optimize conditions for GAD system activation in *Lv. brevis* CRL 2013. While further studies will be required to identify the specific sensing and regulatory elements involved, the present dataset provides a coherent basis for future mechanistic work.

## 4. Materials and Methods

### 4.1. Microorganisms, Culture Media, and Growth Conditions

*Lv. brevis* CRL 2013, belonging to the CERELA culture collection, was routinely grown in modified MRS broth (mMRS, Biokar Diagnostics, Allonne, France) containing 10 g/L fructose and 10 g/L glucose (MRS-GF) at 30 °C for 16 h. Cells were harvested by centrifugation (8000× *g*, 15 min), washed twice with sterile 0.80% NaCl solution, and resuspended in this saline solution to the original volume. This suspension was used to inoculate a chemically defined medium (CDM) at an initial optical density at 600 nm (OD600) of 0.1 (≈5 × 10^7^ CFU/mL).

CDM was prepared according to [20] and contained (g/L) glucose, 10; fructose, 10; KH_2_PO_4_, 3; K_2_HPO_4_, 3; sodium acetate, 5; ammonium citrate, 1; MgSO_4_·7H_2_O, 0.2; MnSO_4_·4H_2_O, 0.025; L-alanine, 0.3; L-arginine, 0.3; L-asparagine, 0.6; L-aspartic acid, 0.6; L-cysteine, 0.5; L-glutamine, 0.8; L-glutamic acid, 0.8; glycine, 0.3; L-histidine, 0.3; L-isoleucine, 0.2; L-leucine, 0.2; L-lysine, 0.3; L-methionine, 0.3; L-phenylalanine, 0.3; L-proline, 0.3; L-serine, 0.3; L-threonine, 0.3; L-tryptophan, 0.2; L-tyrosine, 0.2; L-valine, 0.2; uracil, 0.01; guanine, 0.01; adenine, 0.01; nicotinic acid, 0.001; calcium pantothenate, 0.001; pyridoxal, 0.002; thiamine, 0.001; folic acid, 0.001; cyanocobalamin, 0.001; riboflavin, 0.001; and Tween 80 (1 mL/L). All compounds were of analytical grade (Sigma-Aldrich, St. Louis, MO, USA). Media were adjusted to pH 6.5 and sterilized by filtration through 0.2-µm pore size sterile filters (Gelman Sciences, Ann Arbor, MI, USA).

To evaluate the role of nitrogen sources on GABA production, CDM containing 5% (*w*/*v*) monosodium glutamate (CDMg) as precursor was supplemented with different nitrogen sources (Table S3); 1% (*w*/*v*) yeast extract (Difco Laboratories, Sparks, MD, USA, CDMgYE), Casitone (Difco, 0.5, 1, 2, or 5% *w*/*v*; CDMgC0.5, CDMgC, CDMC2 and CDMC5, respectively), Casamino Acids (Difco 1% *w*/*v*; CDMgCA), Tryptone (Difco, 1% *w*/*v*; CDMgT), or vegetable peptone (Difco, 1% *w*/*v*; CDMgVP). Cultures were incubated at 30 °C and sampled at different time intervals to monitor cell growth (OD600), pH, and GABA production under these conditions. All culture conditions were tested in biological triplicate.

### 4.2. Nitrogen Source Fractionation and Peptide Supplementation Assays

To evaluate whether low-molecular-mass peptides contribute to GAD system activation, Casitone (Difco) was fractionated into >3 kDa (high-molecular-mass peptide fraction, HMMP) and <3 kDa (low-molecular-mass peptide fraction, LMMP). Fractionation was performed by centrifugal ultrafiltration at 3000× *g* using 3 kDa-cutoff Centricon^®^ membranes (Amicon, Beverly, MA, USA). The LMMP fraction was collected as the filtrate, while HMMP was recovered from the retentate.

### 4.3. GABA Measurements

GABA was quantified using a modified version of the GABase method [16]. NADPH formation was monitored spectrophotometrically at 340 nm every 1 min for 10 min at 25 °C in a Biotek Synergy HT microplate reader (Winooski, VT, USA). GABA concentration in each sample was calculated from a calibration curve prepared with standard solutions (0.1, 0.25, 0.5, and 1.0 mM GABA).

### 4.4. Proteomic Analysis

Proteomic analysis was performed according to the methodology described by [39], with minor modifications. Cells of *Lv. brevis* CRL 2013, grown for 36 h in CDMg, CDMgC, and CDMgYE media, were harvested by centrifugation (8000× *g*, 10 min, 4 °C). The resulting pellets were stored at −80 °C until further analysis.

#### 4.4.1. Sample Preparation

Intracellular proteins were analyzed using a shotgun bottom-up proteomics strategy with a label-free data-dependent acquisition (LFQ-DDA) approach. Cells were washed three times with 50 mM phosphate buffer (pH 7.0) and disrupted by bead beating in the presence of PMSF and EDTA. After centrifugation, cell debris and beads were removed, and the protein concentration was determined using the Bradford method (Bio-Rad Laboratories Inc., Hercules, CA, USA). A total of 30 µg of protein per sample was reduced with dithiothreitol, alkylated with iodoacetamide, and digested overnight with trypsin. Peptides were extracted with acetonitrile, dried with a SpeedVac, resuspended in 0.1% trifluoroacetic acid, and desalted using ZipTip C18 columns (Merck KGaA, Darmstadt, Germany).

#### 4.4.2. LC-MS/MS Data Acquisition

Digested peptides were analyzed by nano-HPLC using an EASY-nLC 1000 chromatograph coupled to a Q-Exactive HF mass spectrometer (Thermo Scientific, Waltham, MA, USA) with an Orbitrap analyzer and HCD fragmentation. Full MS scans were acquired at 70,000 resolution, followed by MS/MS of the 12 most intense ions per cycle at 17,500 resolution. The mass tolerance was set to 10 ppm, and a dynamic exclusion list was applied to minimize the repeated sequencing of the same peptides.

#### 4.4.3. Protein Identification and Quantification

Spectra were analyzed with Proteome Discoverer 2.2 (Thermo Scientific) against the *Lv. brevis* ATCC 367 reference proteome (UniProtKB). Proteins identified only by site, reverse hits, or contaminants were removed. The search parameters included: two missed cleavages, carbamidomethylation of cysteine as a fixed modification, oxidation of methionine, and phosphorylation (Ser/Thr/Tyr) as variable modifications, and a minimum peptide length of six amino acids. Protein identification required at least two peptides, with one unique, and was validated at a 1% FDR using a target-decoy strategy. The mass spectrometry proteomics data have been deposited to the ProteomeXchange Consortium via the PRIDE partner repository with the dataset identifier PXD068495.

#### 4.4.4. Proteomic Data Analysis

Protein quantification and statistical analyses were performed using Perseus v1.6.14 [40]. Label-free quantification (LFQ) intensity values were log2-transformed, and missing values were imputed from a normal distribution. Proteins exclusively present in one condition (i.e., absent in all replicates of the compared condition) were retained if they met the minimum valid value criteria. To assign biological functions and categorize the identified proteins, we first converted protein accession numbers to gene names, Gene Ontology (GO) terms, and metabolic pathways using UniProt ID Mapping [41]. We then submitted the FASTA sequences of the protein set to EggNOG mapper [42] to obtain functional categories, which were used for subsequent analyses. Differentially expressed proteins (DEPs) were identified using a two-sample Student’s *t*-test with Benjamini–Hochberg false discovery rate (FDR) correction (FDR < 0.05) for each pairwise comparison (CDMg vs. CDMgC, CDMg vs. CDMgYE, CDMgC vs. CDMgYE). Fold changes ≥ 2 were required for DEP classification. This FDR-corrected *t*-test approach follows standard LFQ proteomics workflows implemented in Perseus. Principal component analysis (PCA) was performed in Perseus, while hierarchical clustering, Venn diagrams of DEPs, pie charts of functional categories, and bar plots of the most differentially expressed proteins were generated in Jupyter Notebook (Python, version 3.9; https://jupyter.org). To visualize the overlap of DEPs between conditions, a Venn diagram was generated from the sets of significant proteins obtained from the FDR-corrected pairwise comparisons, allowing identification of condition-specific and shared expression patterns.

### 4.5. Real-Time Quantitative PCR

#### 4.5.1. Total RNA Extraction and cDNA Synthesis

Total RNA was extracted using the Macaloid Clay method [19]. The RNA concentration was quantified spectrophotometrically using a Nabi-UV/Vis Nano Spectrophotometer (MicroDigital Co., Seoul, Republic of Korea). Genomic DNA was removed from the RNA samples with 5 U of TURBO™ DNase (Life Technologies, Carlsbad, CA, USA), and the absence of residual genomic DNA was confirmed by PCR.

Complementary DNA (cDNA) was synthesized from 1 µg of total DNA-free RNA. The synthesis was carried out using random hexamer primers and LunaScript RT SuperMix Kit (New England Biolabs, Ipswich, MA, USA) according to the manufacturer’s instructions.

#### 4.5.2. qPCR Assay

RT-qPCR was performed on an iQ5 Real-Time PCR Detection System (Bio-Rad Laboratories Inc., Hercules, CA, USA) in 96-well plates. cDNA samples were used as templates, with amplification products detected using the SYBR Green fluorophore (contained in the iQTM SYBR^®^ Green Supermix Kit, Bio-Rad Laboratories Inc.). Each reaction was carried out in triplicate in a final volume of 20 μL containing: 10 μL of 2X iQTM SYBR^®^ Green Supermix, 200 nM of each primer (Table S4), and 30 ng of cDNA.

The thermal cycling conditions were: an initial denaturation at 95 °C for 5 min, followed by 40 cycles of: 95 °C for 1 min, 55 °C for 1 min, and 72 °C for 30 s. A subsequent melting curve analysis (81 cycles of 10 s starting at 55 °C) was performed to monitor the dissociation kinetics of the amplified fragments. Chromosomal DNA and RNA were used as positive and negative control templates, respectively. A non-template control (NTC) was also included in the analysis. The relative expression of the analyzed genes was quantified using the resulting PCR efficiency and the initial fluorescence (N_0_). The amplification curves were analyzed using LinRegPCR software (version 2021.2.0.0) to determine the real efficiency of each reaction, avoiding assuming an ideal efficiency of 100% [43]. The average of these efficiencies is calculated and used for each amplicon. This efficiency is incorporated into the relative expression calculation to reduce potential biases. The geometric mean of the housekeeping *recA* and *rpoD* genes was used to normalize the values obtained for each sample, which improves the robustness and precision of normalization.

### 4.6. Statistical Analysis

For physiological, biochemical (growth, pH, and GABA production), and transcriptional data, statistical analyses were performed using GraphPad Prism 8 (GraphPad Software Inc., San Diego, CA, USA). Data were analyzed by one-way ANOVA followed by Tukey’s post hoc test, with a significance level set at *p* < 0.05. Results are expressed as mean ± standard deviation (SD) of three independent biological replicates.

Multivariate analyses were performed in Perseus (v1.6.14) using log2-transformed and z-score normalized data. Principal component analysis (PCA) was applied to reduce dimensionality and visualize global variance structure. Statistical support for group separation was assessed using permutational multivariate analysis of variance (PERMANOVA, Euclidean distance, 999 permutations), analysis of similarities (ANOSIM), and permutational analysis of multivariate dispersion (PERMDISP). Significance was set at *p* < 0.05.

For proteomic data, statistical and bioinformatic analyses were carried out in Perseus v1.6.14 [40]. LFQ intensities were transformed into log2, and missing values were imputed from a normal distribution. Proteins were retained if identified by at least two unique peptides and present in all replicates of at least one experimental group. DEPs were identified using a two-sample Student’s *t*-test with Benjamini–Hochberg false-discovery-rate (FDR) cutoff of 0.05 and a log2 fold change ≥1 (upregulated) or ≤−1 (downregulated). PCA was performed in Perseus, while hierarchical clustering, Venn diagrams of DEPs, pie charts of functional categories, and bar plots were generated in Jupyter Notebook (Python version 3.9; https://jupyter.org).

## 5. Conclusions

GABA biosynthesis in *Lv. brevis* CRL 2013 is strongly influenced by the composition and concentration of the nitrogen source, with peptide-rich inputs playing a central role. Our integrated physiological, proteomic, and transcriptional analyses showed that peptide availability is associated with an induction of the *gadRCB-gltX* operon and a marked increase in GadB abundance, in parallel with broader metabolic adjustments that support active GABA synthesis. These findings highlight GadB levels as a key determinant of GABA synthesis and emphasize that peptide complexity, not simply total nitrogen, shapes the magnitude of the response. This knowledge provides a framework for optimizing functional food fermentations by considering the peptide composition of raw materials to enhance and better control GABA production in fermented matrices.

## Figures and Tables

**Table 1:** Effect of nitrogen source supplementation on cell growth, final pH, and GABA production in a chemically defined medium (CDM) containing monosodium glutamate (CDMg) by *Lv. brevis* CRL 2013 after 72 h of incubation.

Medium ^1^	Specific Growth Rate (h^−1^)	Final Cell Density (OD 600 nm)	GABA Production (mM)	Final pH
CDMg	0.20 ± 0.02 ^a^	1.60 ± 0.12 ^a^	ND	4.90 ± 0.05 ^c^
CDMYE	0.34 ± 0.02 ^c^	6.19 ± 0.18 ^d^	ND	4.69 ± 0.04 ^d,e^
CDMC2	0.33 ± 0.02 ^c^	6.29 ± 0.19 ^d^	ND	4.68 ± 0.05 ^e^
CDMgYE	0.34 ± 0.03 ^c^	5.85 ± 0.22 ^d^	258 ± 12 ^a^	7.24 ± 0.05 ^a^
CDMgC0.5	0.24 ± 0.02 ^a,b^	2.81 ± 0.15 ^b^	ND	4.93 ± 0.04 ^c^
CDMgC	0.28 ± 0.02 ^b^	3.55 ± 0.19 ^c^	156 ± 11 ^b^	6.11 ± 0.03 ^b^
CDMgC2	0.33 ± 0.02 ^c^	6.30 ± 0.19 ^d^	251 ± 17 ^a^	7.10 ± 0.04 ^a^
CDMgC5	0.34 ± 0.02 ^c^	6.35 ± 0.20 ^d^	255 ± 11 ^a^	7.15 ± 0.04 ^a^
CDMgCA	0.26 ± 0.02 ^a,b^	2.71 ± 0.25 ^b^	ND	4.85 ± 0.05 ^c^
CDMgT	0.25 ± 0.03 ^a,b^	2.68 ± 0.18 ^b^	ND	4.83 ± 0.04 ^c,d^
CDMgVP	0.26 ± 0.02 ^a,b^	2.81 ± 0.24 ^b^	15 ± 1 ^c^	4.87 ± 0.04 ^c^
CDMgLMMP	0.25 ± 0.02 ^a,b^	3.45 ± 0.28 ^c^	165 ± 15 ^b^	6.09 ± 0.04 ^b^
CDMgHMMP	0.22 ± 0. 02 ^a,b^	2.91 ± 0.28 ^b^	16 ± 3 ^c^	4.96 ± 0.05 ^c^

^1^ CDMg, chemically defined medium with glutamate; CDMYE, CDM + 1% yeast extract; CDMC2, CDM + 2% Casitone (no glutamate added); CDMgYE, CDMg + 1% yeast extract; CDMgC0.5, CDMg + 0.5% Casitone; CDMgC, CDMg + 1% Casitone; CDMgC2, CDMg + 2% Casitone; CDMgC5, CDMg + 5% Casitone; CDMgCA, CDMg + 1% Casamino Acids; CDMgT, CDMg + 1% Tryptone; CDMgVP, CDMg + 1% Vegetable Peptone; CDMgLMMP, CDMg + 1% low-molecular-mass peptides from Casitone (<3 kDa); CDMgHMMP, CDMg + 1% high-molecular-mass peptides from Casitone (>3 kDa). Results are presented as means ± standard deviations from at least 3 independent biological replicates. The initial pH of all CDM was approximately 6.5. Different letters within a column indicate significant differences according to one-way ANOVA followed by Tukey’s test (*p* < 0.05). ND, not detected.

## Data Availability

The original data presented in the study are openly available in [ProteomeXchange Consortium] at [http://www.ebi.ac.uk/pride] accessed on 18 September 2025 or [PXD068495].

## Supplementary Materials

The following supporting information can be downloaded at: https://www.mdpi.com/article/10.3390/ijms27010082/s1.
