# Supplementary material for: Influence of Peptide-Rich Nitrogen Sources on GAD System Activation and GABA Production in Levilactobacillus brevis CRL 2013
Source: Int J Mol Sci. 2025 Dec 21;27(1):82. doi: 10.3390/ijms27010082 (PMC12785961; doi:10.3390/ijms27010082)
Supplement: Supplementary file 1 [file ijms-27-00082-s001.zip › ijms-4016848-supplementary.pdf]

International Journal of
Molecular Sciences

MDPI

Article

Influence of Peptide-Rich Nitrogen Sources on GAD System Activation and GABA Production in *Levilactobacillus brevis* CRL 2013

María Paulina Urquiza Martínez [1†], Pablo G. Cataldo [1†], Natalia Soledad Rios Colombo [2], Pasquale Ferranti [3], Lucila Saavedra [1*], Elvira M. Hebert [1*]

[1] Centro de Referencia para Lactobacilos (CERELA-CONICET), Chacabuco 145, 4000 San Miguel de Tu-cumán, Argentina. purquiza@cerela.org.ar (M.P.U.M.); pcataldo@cerela.org.ar (P.G.C.)
[2] APC Microbiome Ireland, University College Cork, Cork, Ireland. nsoledadrioscolombo@ucc.ie
[3] Department of Agricultural Sciences, University of Naples Federico II, via Università 100, 80055, Portici, Italy. ferranti@unina.it
* Correspondence: ehebert@cerela.org.ar (E.M.H.); lucila@cerela.org.ar (L. S.)

Table S1. Proteins exclusively detected in peptide-supplemented conditions (CDMgC and CDMgYE) and absent in CDMg.

Protein ID	Locus tag	Annotated function	Protein name	Functional category
Q03RV4	LBR_07030	ACP S-malonyltransferase	FabD	Fatty acid biosynthesis
Q03Q29	LBR_04255	Microcompartment protein	PduB	Bacterial microcompartment formation
Q03R97	LBR_12635	Adapter protein	MecA	Protein quality control (Clp-dependent)
Q03RW3	LBR_07075	Biotin carboxyl-carrier protein ligase	BirA	Biotin metabolism / fatty acid synthesis
Q03P45	LBR_02445	Glucanase	Endoglucanase	Cell wall modification

Table S2. Differential expression of peptidases. amino acid and peptide transporters in CDMgC vs CDMg.

Protein ID	*Locus* tag	Annotation	Functional category	Fold (CDMgC/ CDMg)	*p* value
Q03N78	LBR_00530	Aminopeptidase C	Peptidase	5.23	0.0002
Q03RY6	LBR_07200	Dipeptidase	Peptidase	4.01	0.0003
Q03PC0	LBR_02810	Endopeptidase O	Peptidase	3.99	0.0065
Q03TP3	LBR_05590	Dipeptidase	Peptidase	2.66	0.0381
Q03QC3	LBR_09520	Dipeptidase	Peptidase	2.42	0.0235
Q03TY2	LBR_05120	OppA (substrate-binding protein)	Oligopeptide ABC transporter	3.83	0.0143
Q03TX8	LBR_05140	OppD (ATP-binding protein)	Oligopeptide ABC transporter	3.51	0.0339
Q03TX9	LBR_05135	OppF (ATP-binding protein)	Oligopeptide ABC transporter	2.89	0.0145
Q03TY1	LBR_05125	OppB (membrane permease)	Oligopeptide ABC transporter	1.94	0.0234
Q03R66	LBR_10145	Glutamine ABC transporter, permease	Amino acid ABC transporter	4.24	0.0006

Table S3. Chemically defined media (CDM) supplemented with different nitrogen sources used in this study.

Medium	Supplementation	Final concentration (w/v)	Supplier (brand)
CDMg	None (baseline chemically defined medium)	—	—
CDMYE	Yeast extract	1%	Difco Laboratories (MD, USA)
CDMC2	Casitone	2%	Difco Laboratories
CDMg	Monosodium glutamate (MSG)	5%	Sigma-Aldrich
CDMgYE	MSG + Yeast extract	5% + 1%	Difco Laboratories
CDMgC0.5	MSG + Casitone	5% + 0.5%	Difco Laboratories
CDMgC	MSG + Casitone	5% + 1%	Difco Laboratories
CDMgC2	MSG + Casitone	5% + 2%	Difco Laboratories
CDMgC5	MSG + Casitone	5% + 5%	Difco Laboratories
CDMgCA	MSG + Casamino Acids	5% + 1%	Difco Laboratories
CDMgT	MSG + Tryptone	5% + 1%	Difco Laboratories
CDMgVP	MSG + Vegetable peptone	5% + 1%	Difco Laboratories

Table S4. Primers used in this study.

Gene or target (locus)	Predicted function	Primer sequence (5´to 3′)	Amplicon length (bp)
rpoD (LBR_08040)	RNA polymerase sigma factor RpoD	*rpoD Fw* TTGGCCTAGATGATGGTCGG<br>*rpoD Rv* GTTTACTGCGTGATGGGTGC	129
recA (LBR_10325)	Protein RecA	*recA Fw* GCCTTGATTTCCAGTGGTGC<br>*recA Rv*: GTGACATCAATCGGGCTTGC	127
gltX (LBR_00010)	Glutamate--tRNA ligase 1	*gltX Fw:* ATGCGGGACCAACAACGGGCC<br>*gltX Rv:* TGGTTCGCCGGCAAATGGAGGC	137
gadA (LBR_00015)	Glutamate decarboxylase	*gadA Fw*: GTTGATGCTGCCTTTGGTGG<br>*gadA Rv*: GCCAAACAATCCACCCCAAC	145
gadC (LBR_00020)	Glutamate-GABA antiporter	*gadC Fw:* CGGCCTTTGGATTTGTCGTG<br>*gadC Rv:* GGCAAAACGAGCATGACCAC	112
gadR (LBR_00025)	Transcriptional regulator	*gadR Fw:* CGTCGATTCCCATGCTTATTCG<br>*gadR Rv:* GCGGAAATGTAACTGCGGAC	115
gadB (LBR_03025)	Glutamate decarboxylase	*gadB Fw:* GCTTCCGGCGGATTTTACAC<br>*gadB Rv:* CAGATTACCCAGCCGACTCC	134

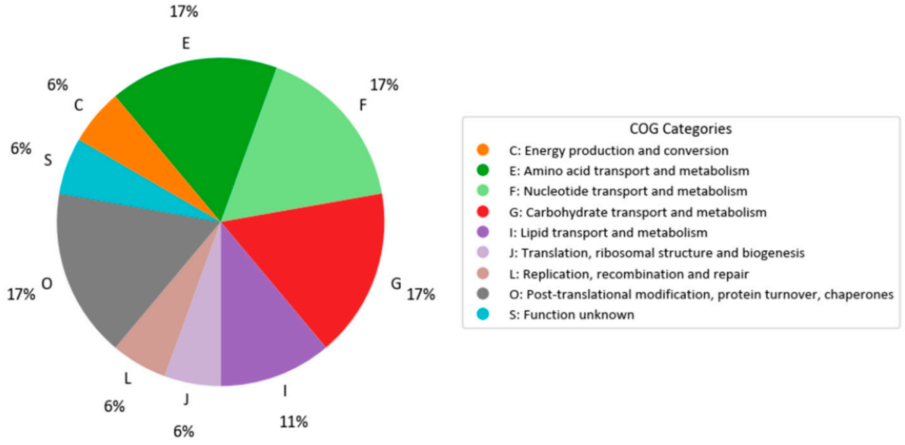

Figure S1. Functional categorization of the proteins shared between CDMgC and CDMgYE and overexpressed relative to CDMg. The pie chart illustrates their distribution across major functional categories, including nucleotide metabolism, fatty acid synthesis, protein quality control, and GABA production. Differentially expressed proteins were identified in Perseus, and functional categorization and visualization were performed in Jupyter Notebook. Within the GABA production category, glutamate decarboxylase (GadB) and glutamyl-tRNA ligase (GltX) were identified, consistent with their coordinated role in GABA biosynthesis. This analysis highlights the metabolic pathways predominantly activated by peptide supplementation, with a stronger induction in yeast extract than in Casitone.
